# Identification of polymorphisms in the bovine collagenous lectins and their association with infectious diseases in cattle

**DOI:** 10.1007/s00251-018-1061-7

**Published:** 2018-05-10

**Authors:** R. S. Fraser, J. S. Lumsden, B. N. Lillie

**Affiliations:** 1 Department of Pathobiology, Ontario Veterinary College, University of Guelph, Guelph, ON Canada; 2 St. George’s University, True Blue, Grenada

**Keywords:** Collagenous lectins, Infectious disease, Cattle, Pooled next-generation sequencing, Genetic variants

## Abstract

Infectious diseases are a significant issue in animal production systems, including both the dairy and beef cattle industries. Understanding and defining the genetics of infectious disease susceptibility in cattle is an important step in the mitigation of their impact. Collagenous lectins are soluble pattern recognition receptors that form an important part of the innate immune system, which serves as the first line of host defense against pathogens. Polymorphisms in the collagenous lectin genes have been shown in previous studies to contribute to infectious disease susceptibility, and in cattle, mutations in two collagenous lectin genes (*MBL1* and *MBL2*) are associated with mastitis. To further characterize the contribution of variation in the bovine collagenous lectins to infectious disease susceptibility, we used a pooled NGS approach to identify short nucleotide variants (SNVs) in the collagenous lectins (and regulatory DNA) of cattle with (*n* = 80) and without (*n* = 40) infectious disease. Allele frequency analysis identified 74 variants that were significantly (*p* < 5 × 10^−6^) associated with infectious disease, the majority of which were clustered in a 29-kb segment upstream of the collectin locus on chromosome 28. In silico analysis of the functional effects of all the variants predicted 11 SNVs with a deleterious effect on protein structure and/or function, 148 SNVs that occurred within potential transcription factor binding sites, and 31 SNVs occurring within potential miRNA binding elements. This study provides a detailed look at the genetic variation of the bovine collagenous lectins and identifies potential genetic markers for infectious disease susceptibility.

**Electronic supplementary material:**

The online version of this article (10.1007/s00251-018-1061-7) contains supplementary material, which is available to authorized users.

## Introduction

Infectious diseases are a major source of morbidity, mortality, and economic loss to the cattle industry. Infectious respiratory diseases alone account for close to $0.5–1 billion USD annually in North America (Miles 2009), and while estimates for other common infectious diseases, such as mastitis and gastrointestinal disease, are difficult to obtain, they undoubtedly add significantly to the economic impact of infectious disease (Schepers and Dijkhuizen 1991; Halasa et al. 2007; Heikkilä et al. 2012). Infectious diseases also represent a large source of agricultural antimicrobial use, which contributes to the development of antimicrobial resistance (Prescott et al. 2012). The approach to managing the impact of infectious disease has traditionally been to control the pathogen, largely ignoring the potential contributions of an immunologically deficient host (Miles 2009). Recently, however, there has been a broadening in focus to include host factors that contribute to infectious disease susceptibility.

The innate immune system represents the first line of defense against infectious diseases. Pattern recognition receptors (PRRs), a key part of the innate immune system, recognize conserved motifs on pathogens called pattern associated molecular patterns (Janeway 1989). The collagenous lectins are a subset of membrane-bound and/or soluble, circulating C-type lectins that function as PRRs, recognizing carbohydrate residues on the surfaces of bacteria, viruses, and fungi. The collagenous lectin family includes the collectins and ficolins, which share structural and functional similarities. Eleven collagenous lectin genes have been identified in cattle, including the genes encoding mannose-binding lectins A and C (*MBL1* and *MBL2*); surfactant proteins A and D (*SFTPA1* and *SFTPD*); collectins (CL) 10, 11, 12, 43, and 46; conglutinin (*CGN1*); and ficolin-1 (*FCN1*). *CL43*, *CL46*, and *CGN1* are found in cattle and a few select herbivores, and structural similarities between these collagenous lectins and *SFTPD* suggest that they are evolutionarily related (Hansen and Holmskov 2002; Gjerstorff et al. 2004a).

Certain collagenous lectins can activate the lectin pathway of complement and can agglutinate or opsonize pathogens (Fujita 2002, 2004). The lectin pathway of complement is activated in part by four MBL-associated serine proteases (MASPs), encoded by two MASP genes, *MASP1* and *MASP2*. The MASP proteins are structurally and functionally similar to C1r and C1s of the classical complement pathway (Thiel et al. 1997; Matsushita et al. 2013) and bind in proenzyme form to the collagen-like domain of the MBLs, FCN-1, and CL-11 (Matsushita and Fujita 1992; Matsushita et al. 2001; Hansen et al. 2010). Following ligand recognition, the MASP proteins lead to the cleavage of complement components C2 and C4, resulting in the activation of complement.

Short nucleotide variants (SNVs) in the collagenous lectin genes are associated with infectious disease susceptibility in a variety of species. A dominant negative missense mutation in human *MBL2* leads to an opsonic defect and is a cause of recurring infections in children (Sumiya et al. 1991) and adults (Summerfield et al. 1995). Deficiencies of MBL-C resulting from *MBL2* polymorphisms in humans are also associated with HIV, hepatitis B and C, meningococcal disease, and parasitic infections (Eisen and Minchinton 2003). Polymorphisms in the human ficolin genes are associated with leprosy (Boldt et al. 2013; Andrade et al. 2017), pneumonia (van Kempen et al. 2017) and Chagas disease (Luz et al. 2016), while variation in *SFTPA2* is associated with different outcomes to influenza A virus infection (Herrera-Ramos et al. 2014). In animals, mutations in the promoter region of porcine *MBL2* are associated with decreased expression of MBL-C and are more frequent in animals diagnosed with pneumonia, enteritis, serositis, or septicemia (Lillie et al. 2007). A missense mutation in porcine *MBL1* is associated with decreased serum concentrations of MBL-A (Juul-Madsen et al. 2011). Relatively little is known about genetic variation in bovine collagenous lectins, and the few investigations that have been done have focused solely on mastitis and the *MBL* genes. Both a missense mutation in exon 2 and a promoter mutation of *MBL1* are associated with altered activity of the classical complement pathway as well as with somatic cell score (SCS), a measure of the inflammatory cell content of milk and an indicator of mastitis (Wang et al. 2011; Liu et al. 2011; Yuan et al. 2012). Mutations in the coding region of *MBL2* are also associated with SCS and complement activity (Zhao et al. 2012; Wang et al. 2012).

In order to address this gap in knowledge, we designed a targeted, next-generation sequencing study that captured the bovine collagenous lectin and related MASP genes as well as surrounding regulatory DNA. We sequenced our target regions in 120 cattle, 80 of which were diagnosed with infectious disease, and 40 of which lacked any evidence of infectious disease. We provide a comprehensive look at the variation in the collagenous lectin genes of these cattle, including in silico predictions of functional effects of identified variants. We also performed association analysis and identified 74 variants significantly associated with infectious disease in cattle.

## Materials and methods

### Sample selection and library preparation

Samples of liver or lung were collected from cattle presenting to the postmortem service at the Ontario Veterinary College or the Animal Health Laboratory at the University of Guelph. Cattle underwent a complete autopsy under the supervision of a veterinary pathologist certified by the American College of Veterinary Pathologists. Ancillary testing (e.g., bacterial culture, viral PCR, etc.) was performed as necessary to confirm the presence of pathogens. Cattle were broadly divided into two major populations: those with and without evidence of infectious disease (referred to as infectious and noninfectious in this article). They were then subdivided into pools of five animals each based on the similarity of their diagnosis at autopsy (Table 1). The study population consisted predominantly of female Holstein-Friesians (66.7%), with fewer intact and castrated male Holstein-Friesians (8.3%). The remainder (25.0%) was composed of a variety of other breeds and crosses (Table 2).

**Table 1:** Cattle were placed into groups of five animals each based on the similarity of the diagnosis determined at autopsy

Status	Group	Diagnosis
Noninfectious	Group 1	Normal (no lesions)
	Group 2	Fractures or trauma
	Group 3	Dental malocclusion, peripheral neuropathy
	Group 4	Intestinal accident or musculoskeletal trauma
	Group 5	Neoplasia
	Group 6	Metabolic disease
	Group 7	Congenital malformations
	Group 8	Organ torsion or rupture
Infectious	Group 9	Endocarditis
	Group 10	Meningitis
	Group 11	Bronchopneumonia 1
	Group 12	Bronchopneumonia 2
	Group 13	Pneumonia (*M. haemolytica*)
	Group 14	Mycoplasma arthritis, osteomyelitis, or pneumonia
	Group 15	Pneumonia (*T. pyogenes*)
	Group 16	Sepsis
	Group 17	Omphalophlebitis
	Group 18	Foot abscess or ulcer
	Group 19	Infectious arthritis
	Group 20	Abortion or perinatal death of infectious cause
	Group 21	Diarrhea
	Group 22	Mastitis
	Group 23	Multifocal abscesses
	Group 24	Metritis or endometritis

**Table 2:** Breakdown of the study population by breed and gender

	Breed	F	M	MN	Total
Noninfectious	Holstein-Friesian	30	1	2	33
	Limousin	1	1		2
	Hereford-Limousin	1			1
	Limousin cross	1			1
	Not available	1			1
	Angus		1		1
	Jersey		1		1
Subtotal		34	4	2	40
Infectious	Holstein-Friesian	50	7		57
	Limousin	2	2	1	5
	Shorthorn	1	1		2
	Red Angus	1			1
	Wagyu	1			1
	Black Angus	1	3	2	6
	Simmental cross	1			1
	Unspecified beef breed	2			2
	Angus		1	1	2
	Angus-Simmental		1		1
	Charolais cross			1	1
	Jersey		1		1
Subtotal		59	16	5	80
Total		93	20	7	120

*MN* male neutered (steer)

Tissue samples were stored at − 20 °C until processed. DNA was extracted using a commercial column based DNA extraction kit (QIAGEN DNeasy Blood and Tissue kit, Mississauga, ON, Canada), and sample concentration was evaluated via fluorometry (Qubit 2.0, Thermo Fisher Scientific, Mississauga, ON, Canada). Equimolar amounts of DNA from cattle in each group were pooled to obtain a final concentration of 1 μg of DNA in 50 μl of low-EDTA buffer TE. Each pool of DNA was acoustically sheared to a target range of 600 bp (Covaris M220, Woburn, MA, USA).

Following acoustic shearing, each pool of DNA underwent end repair, A-tailing, and adapter ligation (including a unique index) using a KAPA Library Preparation Kit for Illumina Platforms (KAPA Biosystems, Wilmington, MA, USA) as per the manufacturer’s instructions, with the following exception: a single cleanup step was performed following adapter ligation. The cleanup was performed by adding 0.6X PEG/NaCl SPRI solution and only the DNA bound to the magnets was retained. The pools were then combined in equimolar amounts to create a single sequencing library.

Target enrichment was performed using a SeqCap EZ Developer Enrichment Kit as per the SeqCap EZ Library SR User’s Guide v.4.2. Target regions consisted of the collagenous lectins and related *MASP* genes and were based on coordinates obtained from the UMD3.1.1 (bosTau8) genome (The Bovine Genome Sequencing and Analysis Consortium et al. 2009) hosted by the University of Santa Cruz, CA (Karolchik et al. 2004) (Table 3). Up to 50 kb upstream and 3 kb downstream of each gene was targeted for sequencing, in an attempt to capture a portion of regulatory DNA. Due to some inconsistencies in the annotation of the bovine collagenous lectins, annotations from NCBI and Ensembl were compared and reviewed, and the most appropriate annotation for each gene was chosen. For example, the *MBL1* gene is not annotated in Ensembl or UCSC, while the *SFTPA1* gene, located nearby on the same chromosome, has four annotated transcript variants in Ensembl. The NCBI RefSeq accession for *MBL1*, NM_001010994.3, is identical to the Ensembl *SFTPA1* transcript ENSBTAT00000001165, leading to some uncertainty surrounding the true identity of these transcripts. Alignment of the four bovine *SFTPA1* transcripts to the coding and protein sequences *SFTPA1* and *MBL1* from other species using Clustal Omega (Goujon et al. 2010; Sievers et al. 2011) showed that ENSBTAT00000031298 and ENSBTAT00000001165 had the highest percent identity matrices to *SFTPA1* and *MBL1*, respectively. Thus, in contradiction to the annotation found in Ensembl, ENSBTAT00000001165 was considered to represent the bovine *MBL1* gene, while only the ENSBTAT00000031298 transcript was considered to represent the *SFTPA1* gene. Similarly, we believe the gene annotated as *FCNB* in Ensembl and *FCN1* in NCBI more closely resembles *FCN1*, and is referred to as such in this study. Coordinates for *COLEC10* and *MASP2* were adjusted slightly based on sequence homology to other species in order to ensure they contained start codons. *CL43* was adjusted to correspond to the findings of Hansen et al. (2003). A complete list of the coordinates for gene annotation in this study is provided in Online Resource 1.

**Table 3:** The regions targeted for resequencing are given for each gene included in the study. Genes in close proximity to each other were sequenced as a single unit

Name	Ensembl ID	Chr	Target start	Target end	Total bp
*MASP1*	ENSBTAG00000012467	1	80,546,924	80,652,367	105,443
*COLEC11*	ENSBTAG00000016225	8	112,860,707	112,896,491	35,784
*FCN1*	ENSBTAG00000048155	11	106,773,026	106,834,643	61,617
*COLEC10*	ENSBTAG00000017343	14	47,260,662	47,359,061	98,399
*MASP2*	ENSBTAG00000012808	16	43,449,518	43,481,362	31,844
*COLEC12*	ENSBTAG00000007705	24	35,627,928	35,866,269	238,341
*MBL2*	ENSBTAG00000007049	26	6,294,785	6,351,912	57,127
*CGN1*	ENSBTAG00000006536	28	35,541,900	35,726,104	184,204
*CL46*	ENSBTAG00000048082	28			
*CL43*	ENSBTAG00000047317	28			
*SFTPD*	ENSBTAG00000046421	28	35,764,587	35,870,565	105,978
*MBL1*	ENSBTAT00000001165^a^	28			
*SFTPA1*	ENSBTAT00000031298^a^	28			

^a^Both of these transcript IDs are from the gene ENSBTAG00000023032 (*SFTPA1*). The transcript given for *MBL1* is identical to the NCBI RefSeq accession for *MBL1* (NM_001010994.3), and percent identity matrices comparing the coding and protein sequences of these accessions to *MBL1* and *SFTPA1* in other species supports their identities as we have determined them based on in silico analysis

Following enrichment, the library was sequenced using MiSeq Reagent Kit v3 (600-cycle) sequencing chemistry on an Illumina MiSeq (San Diego, CA, USA). In order to achieve adequate depth of sequencing, the same library was sequenced twice. All statistical analyses were performed in R unless otherwise specified (R Core Team 2017).

### Bioinformatic analysis of NGS data

**Fig. 1:**
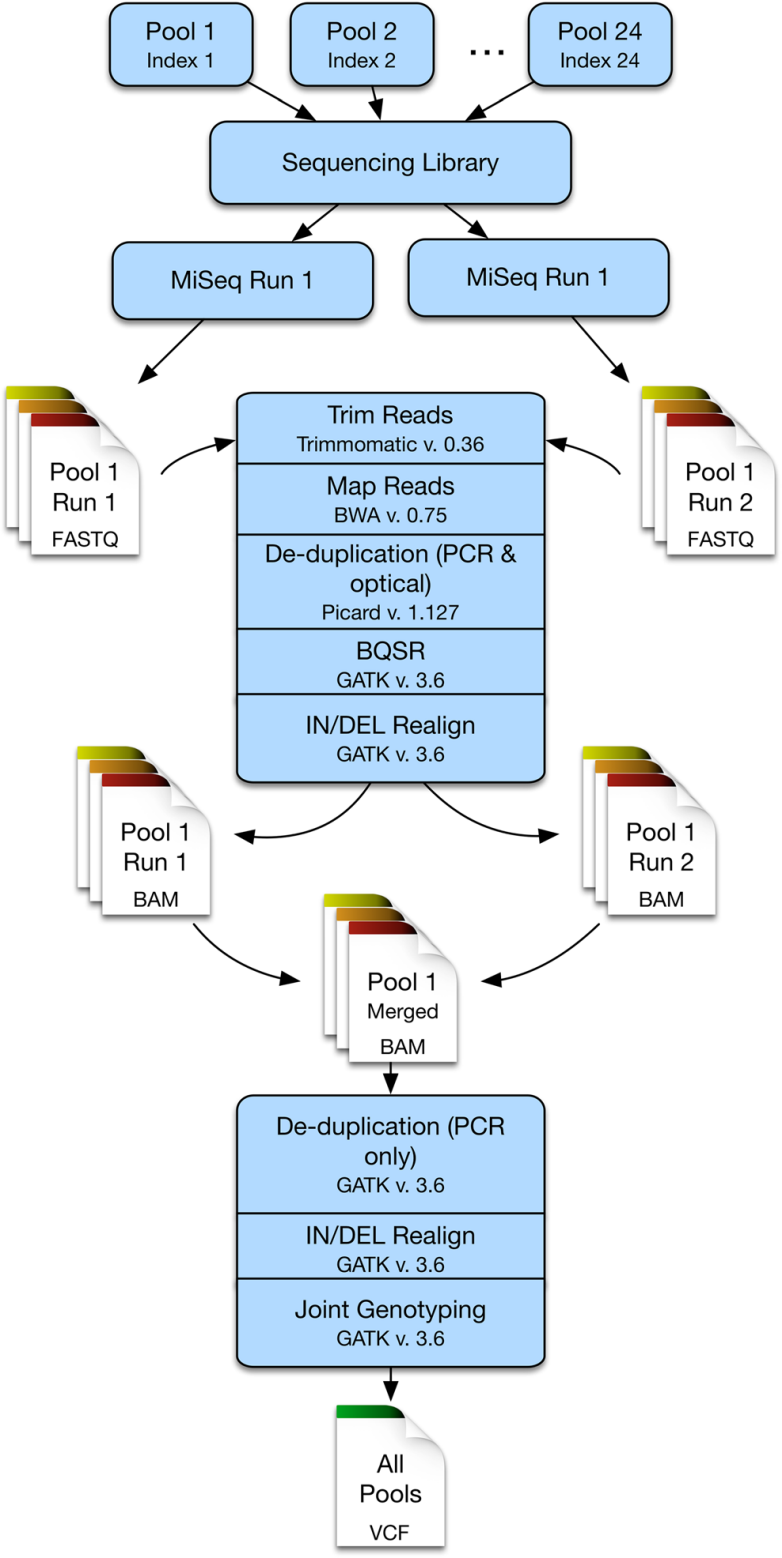
Outline of the bioinformatic steps used to call variants following the two sequencing runs

The sequencing data was processed in two stages (Fig. 1). In the first stage, data from each run was processed separately, while in the second stage, data from the same pool from different runs was merged and then further processed. The data from both sequencing runs was first trimmed using Trimmomatic v. 0.36 (Bolger et al. 2014) based on the following criteria: (a) leading and trailing bases with a quality score of less than 20 were trimmed, (b) reads were trimmed if quality dropped below an average score of 20 over a 5 bp sliding window, and (c) reads were dropped if they were less than 75 bp in length. Reads were then mapped to the bovine genome UMD3.1.1 using BWA-MEM algorithm of BWA v. 0.75 (Li and Durbin 2009). PCR and optical duplicates were removed with Picard v. 1.127 (http://broadinstitute.github.io/picard/, accessed 2018-01-12). The Genome Analysis Toolkit (GATK) Best Practices Guidelines (DePristo et al. 2011; Van der Auwera et al. 2013) were followed for in/del realignment and base quality score recalibration (BQSR) using GATK v. 3.6 (McKenna et al. 2010). At this point, BAM files for each pool generated during the different runs were merged. Each merged BAM file was reprocessed for PCR duplicates and in/del realignment. Variant calling was performed on merged BAM files using the joint genotyping protocol outlined in the GATK Best Practices guidelines. Variants were filtered using separate hard filters for SNVs and in/dels. Multiallelic variants and spanning deletions were excluded, and known variants were obtained from dbSNP v. 150 (Sherry et al. 2001). Evaluation of target capture between the noninfectious and infectious populations was performed by comparing the overall mean of the mean of each pool within each population using a two-way ANOVA and a least-square means post-hoc test.

### Variant analysis

In silico analysis of the variants was performed for coding region variants, downstream 3 kb, and the upstream 5 kb of potential regulatory DNA. Variant density was evaluated both in terms of target regions and functional genomic regions using a one-way ANOVA and Kruskal-Wallis post hoc test. Correlations between variant density and GC content as well as indel number were assessed using a Pearson’s correlation coefficient. Missense coding variants were analyzed using Polyphen2 (Adzhubei et al. 2010) and the SIFT algorithm (Sim et al. 2012) run by the Variant Effect Predictor (VEP) hosted by Ensembl (McLaren et al. 2010). For Polyphen2, batch submission was used and a FASTA file containing the protein sequences was submitted. For VEP, options were left at their default settings.

The 3 kb downstream from the stop codon of the targeted genes was analyzed for potential miRNA recognition elements (MREs). Although the 3′ UTR was annotated within Ensembl for all genes with the exception of *COLEC10* and *SFTPD*, we opted to analyze the entire 3 kb for each gene to maintain consistency. Multiple MRE discovery algorithms were used to maximize the accuracy of the predictions (Riffo-Campos et al. 2016). miRanda v. 3.3a (Enright et al. 2003) and Targetscan v. 7.0 (Agarwal et al. 2015) were used to identify MREs binding mature miR sequences from cattle accessed from miRbase 21 (Kozomara and Griffiths-Jones 2013). The energy threshold parameter of miRanda was set to − 20 kcal/mol, with other parameters for both programs left at their default settings. The intersection of the seed region of predicted MREs from both programs was obtained using BEDtools v. 2.25.0 (Quinlan and Hall 2010). BEDtools was then used to identify variants in our dataset that intersected with the seed sequence of MREs predicted by both algorithms.

Transcription factor (TF) binding site (TFBS) analysis was performed on the 5 kb upstream to the start codon for each target gene using CIS-BP v. 1.02 (Weirauch et al. 2014). The species was set as *Bos taurus*, and a motif model of “PWM - LogOdds” with a minimum threshold of 8 was selected; only *cis* acting TFBSs were considered. Variants falling within predicted TFBSs were identified using BEDtools. Many of the TFBSs shared identical sequences and bound TFs belonging to the same family, and were thus collapsed into a single result with results reported by TF family. Putative TFBSs were further refined by identifying conserved 50-bp-long DNA sequence motifs within the 5 kb upstream from the start codon for each gene across eight different species, cattle (UMD3.1.1), pig (Sscrofa10.2), horse (EquCab2), rat (Rnor_6.0), mouse (GRCm38), gorilla (gorGor3.1), chicken (Galgal4), and human (GRCh38), using MEME v 4.10.1 (Bailey and Elkan 1994). The bovine specific genes *CGN*, *CL43*, and *CL46* were aligned to the sequences of *SFTPD* from other species, as they are believed to be evolutionarily related (Hansen and Holmskov 2002; Gjerstorff et al. 2004b). A minimum E-value of 0.05 was used to consider a motif conserved. The conserved motifs were then examined for the presence of TFBSs predicted by CIS-BP and containing variants.

### Allelic association

The estimated frequency of variant alleles was compared between the noninfectious and infectious populations using Popoolation2 (Kofler et al. 2011). Processed BAM files for each pool were combined using Samtools into a single BAM file for each population, and an mpileup file was generated using minimum mapping and base qualities of 20 (Li et al. 2009). Popoolation2 was used to transform the mpileup file into a sync file, which was then down-sampled according to the recommendations of Popoolation2 to 400 reads per population using the method “fraction” to mitigate the impact of variable read depths on statistical testing. Fisher’s exact test was used to determine the significance of allele frequency estimates between the two populations. A minimum of 5% of the reads (20) across both populations combined was required for the allele to be considered in the allele frequency estimation. The Benjamini-Hochberg procedure was used to correct for multiple testing (Benjamini and Hochberg 1995) with adjusted *p* value cutoffs labeled as described in the BADGE system (Manly 2005).

## Results

**Fig. 2:**
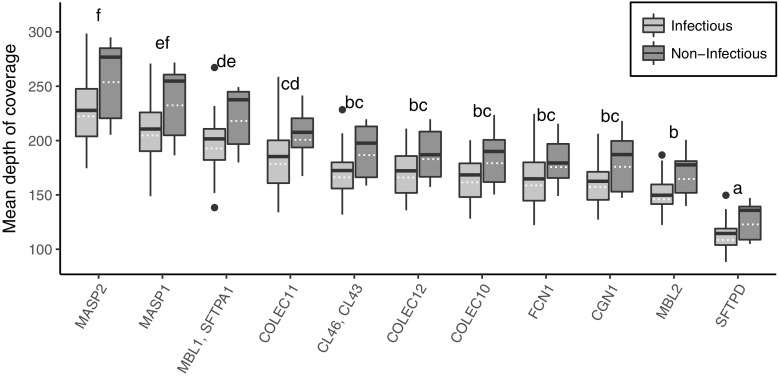
The overall mean of the mean depth of coverage for each pool in the noninfectious and infectious populations by gene target. There were no significant differences between populations in a gene target; however, there was a significant difference in depth of coverage between different gene targets (two-way ANOVA and least-squares means post hoc comparison, *p* < 0.05). Genes that share a letter are not significantly different: for example, *MASP2* is not significantly different from *MASP1* but is significantly different from the remaining gene targets. Solid line represents the median, dashed line is the mean. The hinges represent the 1st and 3rd quantiles, while the whiskers represent 1.5× the interquartile range. Data points beyond this range are illustrated as solid circles

Evaluation of target capture efficiency showed that the median of the mean target coverage was 172.5; however, there were significant differences (*p* < 0.05) both in terms of the depth of coverage for each target region, as well as the total depth of coverage between the noninfectious and infectious populations (Fig. 2). The depth of coverage between the two populations at individual target regions was not statistically different.

Joint genotyping identified a total of 5439 unique variants, all of which were present in both the noninfectious and infectious populations. These included 5418 SNVs and 21 in/dels. The majority (5317, 97.7%) of the variants identified were present in dbSNP 150, while 122 were novel discoveries. A further 32,794 variants were present in dbSNP 150 within our target intervals. Of these, 2922 were present in our population but were excluded due to various filtering parameters, 29,696 loci were not variant in our population, and 356 loci were not successfully sequenced.

**Fig. 3:**
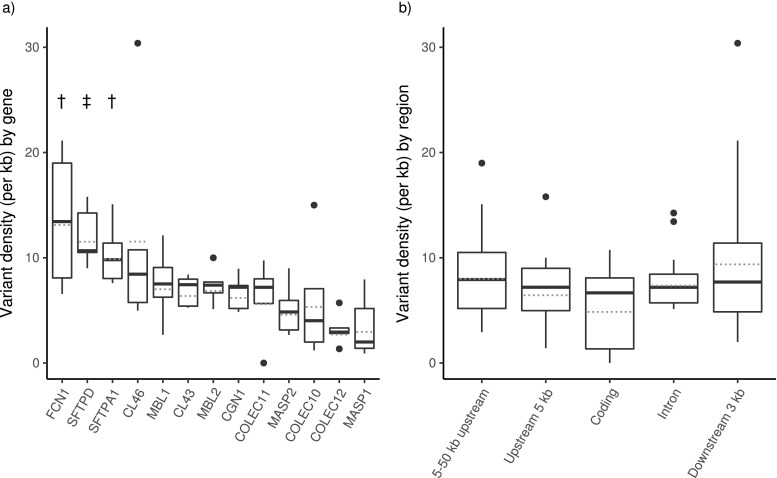
**a** Variant density across the entire study population was significantly different amongst the targeted genes. Variant density in these genes was significantly different than *COLEC12* and *MASP1* (dagger). Variant density was significantly different from *MASP2*, *COLEC10*, *COLEC12*, and *MASP1* (one-way ANOVA and Kruskal-Wallis post hoc test, p < 0.05) (double dagger). **b** Variant density between functional genomic regions was not significantly different. Note that due to the proximity of some genes (e.g., *MBL1* and *SFTPA1*), some variants occurring downstream, upstream, or within the introns and coding regions of *SFTPA1* were also counted as being upstream from *MBL2*, thus, the total of variants by region is different than the total of unique variants discovered. Solid line represents the median, dashed line is the mean. The hinges represent the 1st and 3rd quantiles, while the whiskers represent 1.5× the interquartile range. Data points beyond this range are illustrated as solid circles

A total of 83 coding variants (40 synonymous, 43 missense), 2297 intronic variants, 309 variants within the downstream 3 kb of the stop codon, 414 variants within the 5 kb upstream of the start codon, and 2915 variants in the region between 5 and 50 kb upstream of the start codon were identified. The density of variants was examined both by gene and by region (Fig. 3). Significant differences (*p* < 0.05) in variant density were observed between genes, with the highest density of variants found in the *FCN1* gene and surrounding DNA (Fig. 3a). Variant density by region was not significantly different, though the coding region tended to have the lowest density (Fig. 3b).

Among the coding variants, 43 missense variants were identified. In silico predictions of the effects of the mutations using either algorithm identified a total of 11 SNVs expected to have damaging or possibly damaging effects on protein structure or function (Table 4), with three of the variants (within *SFTPD*, *MBL2*, and *CL43*) predicted to be damaging by both algorithms.

**Table 4:** Potentially deleterious missense mutations as determined by in silico analysis with two different prediction algorithms

Gene	Chr	Pos	rsID	Ref	Alt	Protein change	Protein domain	Polyphen2 Prediction	Polyphen2 Score	SIFT Prediction	SIFT Score
*FCN1*	11	106,827,968	rs382216843	C	T	Arg142Cys	FBG	Probably damaging	0.986	Tolerated	0.06
*MBL2*	26	6,344,919	rs210611099	C	A	Pro42Gln	CLD	Probably damaging	0.974	Deleterious	0
*SFTPD*	28	35,820,078	rs380240341	C	T	Pro132Ser	CLD	Probably damaging	0.958	Deleterious	0.05
*CGN*	28	35,598,640	rs208842091	G	A	Arg173His	CLD	Possibly damaging	0.72	Tolerated	0.16
*CL46*	28	35,675,371	rs383278255	C	T	Pro185Leu	CLD	Possibly damaging	0.672	Tolerated	0.32
*CL43*	28	35,718,034	rs42967143	A	G	Thr117Ala	CLD	Possibly damaging	0.659	Tolerated	0.9
*CL43*	28	35,718,807	rs211678602	G	T	Gln185His	neck region	Possibly damaging	0.634	Deleterious	0.01
*MASP2*	16	43,463,621	rs207667073	G	A	Gly102Ser	CUB domain	Benign	0.191	Deleterious	0.01
*FCN1*	11	106,828,710	rs385211468	C	T	Thr193Met	FBG	Benign	0.042	Deleterious	0.03
*CGN*	28	35,602,463	rs466869949	A	C	Glu302Asp	CRD	Benign	0.036	Deleterious	0.04
*SFTPD*	28	35,824,136	rs110476851	C	G	Ala288Gly	CRD	Benign	0.002	Deleterious	0.02

*FBG* fibrinogen-like domain, *CLD* collagen-like domain, *CUB* complement C1r/C1s, Uegf, Bmp1, *CRD* carbohydrate recognition domain

**Fig. 4:**
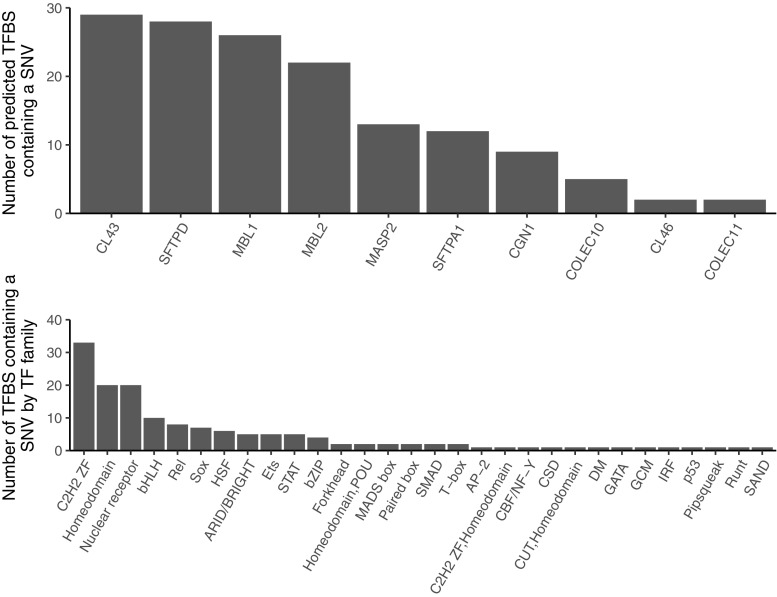
**a** The number of predicted transcription factor binding sites within conserved DNA sequences of the targeted genes that contained a SNV identified in this study. **b** Number of predicted transcription factor families containing a SNV identified in this study

A total of 20,147 potential *cis* TFBSs were predicted in the upstream 5 kb of the targeted genes. Of these, 1351 contained a SNV identified in this study. In highly conserved DNA sequence motifs (based on our multispecies comparison of upstream regulatory regions), 148 TFBSs containing a SNV were found across 10 of the targeted genes (Fig. 4a). These TFBSs were members of 30 TF families (Fig. 4b).

**Fig. 5:**
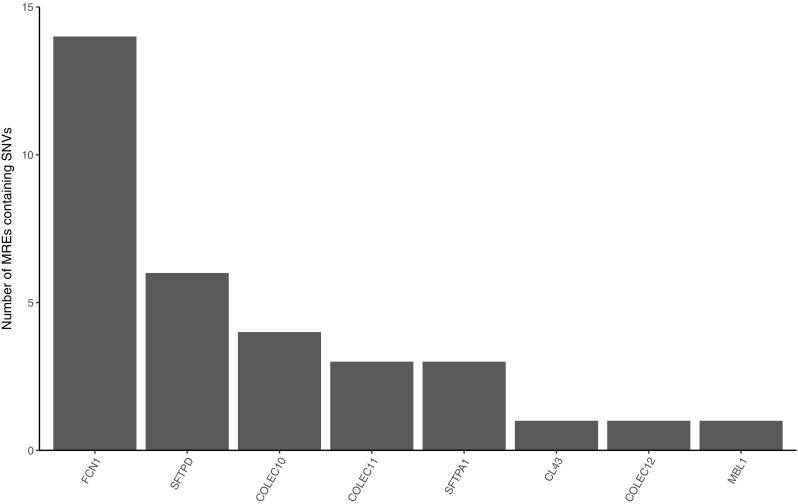
The number of in silico predicted miRNA recognition elements found in the targeted genes

Analysis of the 3 kb downstream from the stop codon using both algorithms identified 469 potential miRNA recognition elements (MREs). Within the seed region of the predicted MREs there were 31 SNVs (Fig. 5), two of which impacted two separate miRs. A total of 28 unique miRs were predicted to bind in these regions, 5 of which bound multiple loci. A single MREs intersected with the annotated 3′ UTR of *SFTPA1*.

**Fig. 6:**
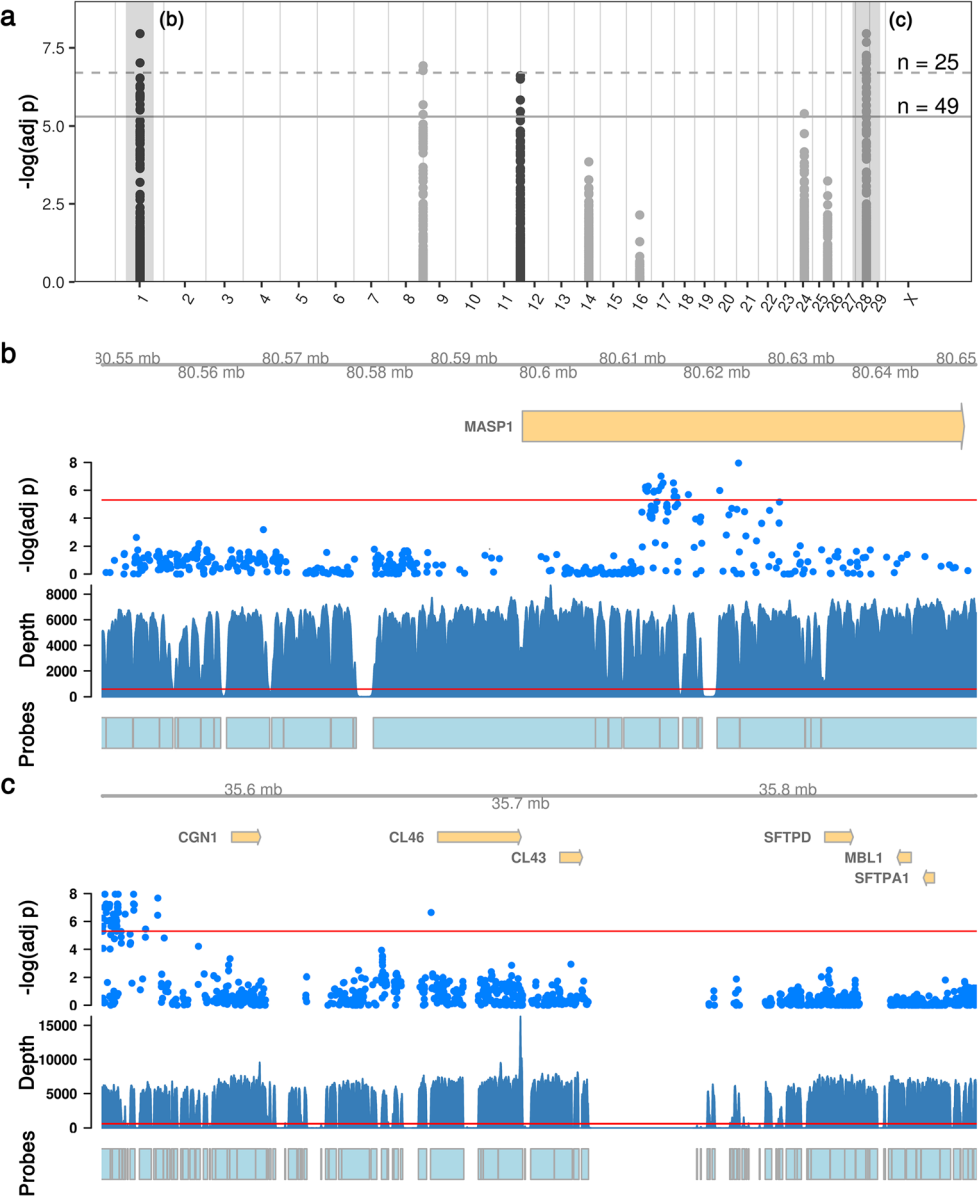
**a** Manhattan plot of the allelic association analysis identified 25 variants significant at BADGE class I and 49 variants at class II (Manly 2005) in 5 different targeted regions. The *p* value has been adjusted for multiple testing using the Benjamini-Hochberg procedure. **b**, **c** The areas highlighted in **a** are shown in greater detail. Significant associations were found within the intron of *MASP1* (**b**) and upstream of the collectin locus (**c**). The red line indicates the cutoff for class II significance (*p* < 5 × 10^−6^). The depth of sequencing (total from all pools) and the regions with probes designed for target capture are shown to illustrate gaps in sequencing and variant discovery

Evaluation of the frequency of variant alleles identified 25 BADGE class I (*p* < 2 × 10^−7^) and 49 class II (*p* < 5 × 10^−6^) variants that were significantly associated with either the Non-infectious or Infectious populations (Fig. 6). Seventeen associations were found clustered in intron 2 of *MASP1* (Fig. 6b), and a further 48 associations were present in a ~ 21 kb region ~ 29 kb upstream from *CGN1*, the first gene of the bovine collectin locus (Fig. 6c). Four associations were found distributed upstream, downstream, and within intron 8 of *FCN1* (Online Resource 2a); four associations were found in the introns 4 and 5 and the downstream region of *COLEC11* (Online Resource 2b); and a single association was found in intron 2 of *COLEC12* (Online Resource 2c).

## Discussion

Associating polymorphisms with infectious disease susceptibility has important implications in agricultural economics, animal breeding, and animal health and welfare. Probing the underlying genetics of complex traits like innate immunity is challenging and requires large numbers of animals with well-defined phenotypes (Ron and Weller 2007). The phenotypes of our study samples were determined through complete autopsies supervised by certified veterinary pathologists. This included evaluation of gross tissues, ancillary testing as required, and review of histopathological specimens to define the extent and nature of disease. In order to address the considerable expense required to sequence large numbers of animals, we opted to use a pooled and targeted next-generation sequence approach. Pooled sequencing has been shown to be an accurate and cost-effective method of variant discovery and allele frequency estimation in next-generation sequencing experiments (Bansal et al. 2011; Mullen et al. 2012; Rellstab et al. 2013; Bertelsen et al. 2016), as well as in genome-wide association studies (Keele et al. 2015). While whole genome sequencing would provide a more complete picture of disease associated variants, the cost remains prohibitive, despite decreasing sequencing costs. Instead, targeted resequencing allows specific regions of interest in the genome to be queried, providing much more detail than the available high density SNP array from Illumina: of the 5439 variants found in our study, only 223 are present in the Illumina BovineHD BeadChip (https://support.illumina.com/array/array_kits/bovinehd_dna_analysis_kit/downloads.html, accessed 2018-01-12).

Allele frequency estimation identified 74 significant variants (BADGE class II or higher), after correction for multiple testing, that were associated with infectious disease. Over half of the significant alleles occurred in a 29-kb segment of DNA upstream from *CGN1*, and these alleles met the highest level of statistical significance proposed by the BADGE system (Manly 2005). *CGN1* is the first gene in the bovine collectin locus, a 260-kb region on chromosome 28 which includes the *CL46*, *CL43*, *SFTPD*, *MBL1*, and *SFTPA1* genes (Gjerstorff et al. 2004a), and several of these genes have been implicated in infectious disease susceptibility. Plasma concentration of conglutinin is known to be heritable and low levels of plasma conglutinin are associated with increased incidence of respiratory disease in cattle (Holmskov et al. 1998). Whole transcriptome sequencing of the abomasum of parasite-susceptible and resistant calves found that resistant animals expressed higher levels of *CL46* (Li et al. 2011). In cattle breeds native to China, certain *MBL1* haplotypes are associated with somatic cell score, an indirect marker of mastitis (Wang et al. 2011). The block of highly significant associations discovered here could theoretically impact any of the genes within the collectin locus, as DNA kilobases to megabases upstream of genes can have a regulatory impact through enhancer, silencer, insulator, or locus control region elements (Maston et al. 2006) that are difficult to predict, and for which the bovine genome lacks annotation. The relative similarity of the allele frequencies in the Non-infectious and Infectious populations was similar for all of these variants, suggesting that they are likely in linkage disequilibrium (Online Resource 3). The pooled design of this study precludes haplotype analysis so further study of these variants, and the genes of the nearby collectin locus, both in terms of RNA expression and epigenetics, is warranted.

Two class I and 15 class II associations were found in intron 2 of *MASP1*. Human *MASP1* encodes three protein isoforms with distinct functions, MASP-1, MASP-3, and MAP1 (Beltrame et al. 2015). Only a single transcript is annotated for bovine *MASP1*, which best corresponds to the MASP-3 isoform, and an additional 4 transcripts are predicted by NCBI. As with human *MASP1*, the transcript and predicted transcripts that encode the three *MASP1* isoforms share the first 8 exons; thus, the cluster of variants noted in intron 2 is also present in intron 2 for all of these predicted transcripts. Genetic mutations resulting in human *MASP1* deficiency are associated with infectious disease, and several intronic mutations leading to *MASP1* deficiency have been identified, though none are identical to the associations found here (Ingels et al. 2013; Beltrame et al. 2015). Again, the relative similarity of the allele frequencies for the different loci suggests significant linkage disequilibrium. It should be noted that mutations that inhibit the function of the MASPs (and complement-activating collagenous lectins) may also confer benefits: excess activation of complement can contribute to tissue damage or autoimmune disease, and decreased or more moderate levels of complement activity may therefore be beneficial in some scenarios (Beltrame et al. 2015).

A further four associations were found in *COLEC11* and two in *COLEC12*; however, no in silico consequences were predicted for any of the six. There is little known about the relative importance of CL-K1 and CL-P1, the proteins encoded by these two genes, in the innate immune response to infectious disease, and, to the authors knowledge, these are the first reported associations between mutations in these genes and infectious disease of cattle. Recently, CL-P1 was shown to have a soluble form that can activate the alternative pathway of complement (Ma et al. 2015), and CL-K1 is capable of activating the lectin pathway of complement (Ma et al. 2013), presenting possible pathways through which genetic mutations could hamper the immune system.

Although in silico analysis of the disease-associated alleles did not identify any biological effects, several interesting predictions were made regarding other variants present in our dataset. Two missense mutations found in *MBL2* and one in *MASP2* (Table 3) were previously shown to be associated with SCS in Chinese Holstein cattle (Wang et al. 2012; Wu et al. 2015). The frequency of these three variants was not significantly different in our populations of infectious and noninfectious cattle. Variant rs210611099:c.125C>A was rare, with only eight alleles predicted across both populations (MAF 3.3%). Though rare, this is substantially higher than the reported allele frequency of 0.27% in Chinese Holstein cattle (Wang et al. 2012), and may be the result of different breeds (Chinese Holsteins versus the mixture of breeds common to North America in our study). The mutation occurs in the collagen-like domain (CLD) of MBL-C and is predicted in silico to have a significant impact on protein structure and function. Previously reported mutations in the CLD had an impact on MBL-C driven complement activation (Larsen et al. 2004), as well as on higher-order oligomerization through disruption of the Gly-X-Y collagen-like repeats (Sumiya et al. 1991). Thus, despite its rarity, the role of this variant in infectious disease susceptibility remains of interest. Genotyping of a larger number of animals may be useful in clarifying the discrepancy between studies in allele frequencies and may provide the statistical power required to determine whether this rare variant plays a role in bovine innate immune defense.

The second *MBL2* missense variant associated with SCS, rs210426415:c.92G>A, leads to the substitution of glutamine for arginine in the N-terminal domain of MBL-C. This domain is believed to utilize conserved cysteine residues to facilitate the functionally critical oligomerization of MBL-C (Wallis and Drickamer 1999). This variant was predicted by both in silico algorithms to have a low impact on protein structure and function, and was not associated with disease in our study, possibly the result of a smaller cohort of animals diagnosed with mastitis. Thus, although genotyping of larger numbers of North American cattle may reveal an association with infectious disease, this study does not provide evidence for further investigation.

The *MASP2* variant previously shown to be associated with SCS, rs207667073.G>A, results in an amino acid change in the CUB domain that is predicted by the SIFT algorithm, but not Polyphen2, to be deleterious to protein structure or function. This allele showed no significant difference between the noninfectious and infectious populations; however, only two alleles were found in the entire population of cattle studied. Again, genotyping of larger number of North American cattle may prove useful in further defining the role of this variant.

Only one variant, rs381773088 in *SFTPA1*, was present within an annotated 3′ UTR and intersected with a predicted MRE for bta-miR-328. To our knowledge, there are no published studies on the role of bta-miR-328 in cattle; however, a study on the human homolog demonstrated that it plays a role in vitro in the innate immune defense against *Haemophilus influenza* through negative regulation of phagocytosis (Tay et al. 2015). Gram-negative pulmonary pathogens related to *H. influenza*, notably *M. haemolytica*, *P. multocida*, and *H. somni*, are important causes of respiratory disease in cattle; thus, this predicted MRE affected by a variant may hold relevance for future investigations.

In silico prediction of transcription factor binding sites relies on the observation that the amino acid sequence of the DNA-binding domain of transcription factor proteins largely predicts their DNA-binding specificity, and does so in a highly conserved manner (Kasahara et al. 2006; Jolma et al. 2013; Weirauch et al. 2014). Regulatory DNA is also highly conserved in animals (Nitta et al. 2015). Thus, to reduce the large number of predicted TFBSs identified in the targeted genes, conserved DNA sequences present in the potential promoter region were identified by comparing sequences from up to eight different animals including domestic livestock and more distantly related species (human, gorilla, cattle, horse, pig, rat, mouse, and chicken). This conservative approach narrowed the results to 148 potential TFBSs, some of which were similar to TFBSs predicted or shown in previous studies to be involved in the regulation of collagenous lectins. For example, we identified binding sites for SRF (a member of the MADS box family of transcription factors) in *SFTPA1*, and an SRF binding site was also identified in human *SFTPA2* (Grageda et al. 2014). Initial characterization of the promoter of *CL43* included in silico prediction of TFBSs (Hansen et al. 2003), and the same binding sites were predicted in our study for Myb, ARNT, cEBP, Myc, MyoD, Mzf-1, N-Myc, and USF; however, only Mzf-1 was both present within a conserved motif and contained a SNV. This discrepancy may be the result of more stringent requirements in our study (including exclusion of *trans* binding sites and sites outside of conserved sequences), or potentially due to differing prediction algorithms. HNF3alpha (also known as FOXA1) is known to regulate the expression of human and chicken *MBL2* (Naito et al. 1999; Kjærup et al. 2013), and while binding sites were predicted in *CL43* and *COLEC11*, no binding site was predicted for bovine *MBL2*.

The density of variants varied significantly by gene, but not by gene region (Fig. 3). *FCN1* and the two surfactant protein genes, *SFTPA1* and *SFTPD*, had a significantly higher variant density than the bottom two (for *FCN1*) and four (for *SFTPA1* and *SFTPD*) genes. Both intrinsic and extrinsic factors can affect the degree of sequence variation. Intrinsically, the GC content and the number of in/dels in a region are predictive of variation (Hodgkinson and Eyre-Walker 2011). The GC content of the *FCN1* gene was the second highest of the targeted genes at 55.1%; however, the overall correlation between GC content and the density of variants was not significant (*R* = 0.36, *p* = 0.23, Online Resource 4). Although the *FCN1* target contained the highest number of indels (13/21), there was no correlation between number of indels and variation density (*R* = 0.36, *p* = 0.23, Online Resource 5). Extrinsically, genes that interact with the environment (such as innate immune response genes) can often be found in “hot spots” in the genome, which show higher levels of variation in response to increased adaptive pressures (Chuang and Li 2004). Interestingly, a recent study on the equine collagenous lectins found that two equine ficolin genes, *FCN1* and *FCN1*-like, also had the highest variant density amongst the equine collagenous lectins (Fraser, unpublished). Five of the eight coding region mutations found in *FCN1* were located within the fibrinogen-like domain, and three were missense. Although the relative role of the ficolin genes in the innate immune response of these two species is still under investigation, the increased variation observed in both species might suggest increased evolutionary pressure to adapt to different pathogens.

Variation in innate immune genes can significantly impact the susceptibility of animals to infectious diseases. Here, we have comprehensively documented the variation in a subset of innate immune genes, the collagenous lectins, in cattle with and without infectious diseases. Comparison of mutant alleles in the two populations identified 74 alleles associated with infectious disease. These alleles warrant further investigation, both in terms of population level frequencies, and, if confirmed to be significantly associated with disease susceptibility, in terms of their biological mechanisms of action.

## Electronic supplementary material

The variants identified in this study have been submitted to the European Variant Archive (EVA) accession PRJEB24763.

ESM 1 (XLSX 28 kb)

ESM 2 (PDF 238 kb)

ESM 3 (XLSX 19 kb)

ESM 4 (PDF 91 kb)

ESM 5 (PDF 88 kb)
