# Supplementary material for: Identification of polymorphisms in the bovine collagenous lectins and their association with infectious diseases in cattle
Source: Immunogenetics. 2018 May 10;70(8):533–46. doi: 10.1007/s00251-018-1061-7 (PMC6061482; doi:10.1007/s00251-018-1061-7)
Supplement: Supplementary file 2 — (PDF 238 kb) [file 251_2018_1061_MOESM2_ESM.pdf]

Electronic Supplementary Data

Identification of polymorphisms in the bovine collagenous lectins and their association with infectious diseases in cattle

Immunogenetics

Russell S. Fraser[1], John S. Lumsden[1,2], Brandon N. Lillie[1]*

[1]Department of Pathobiology, Ontario Veterinary College, University of Guelph

[2]Adjunct Professor, St. George's University, True Blue, Grenada

*Corresponding author: blillie@uoguelph.ca

a)

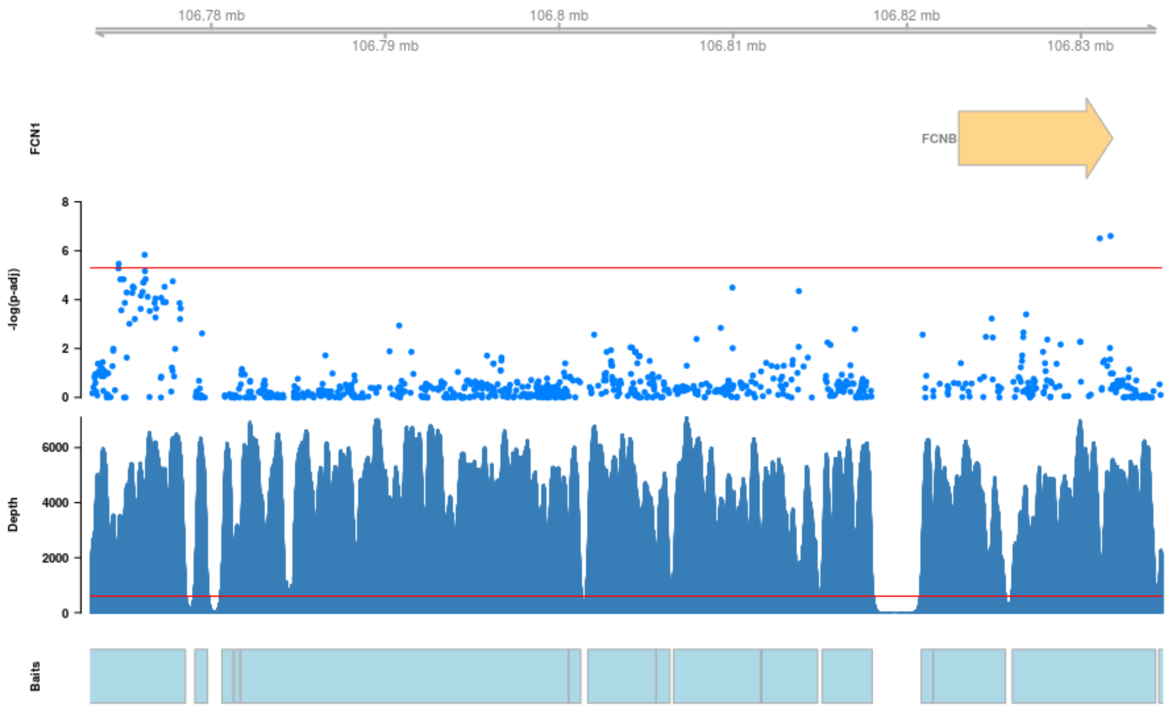
106.78 mb
106.8 mb
106.82 mb
106.79 mb
106.81 mb
106.83 mb
FCN1
FCNB
-log(p-adj)
Depth
Baits

b)

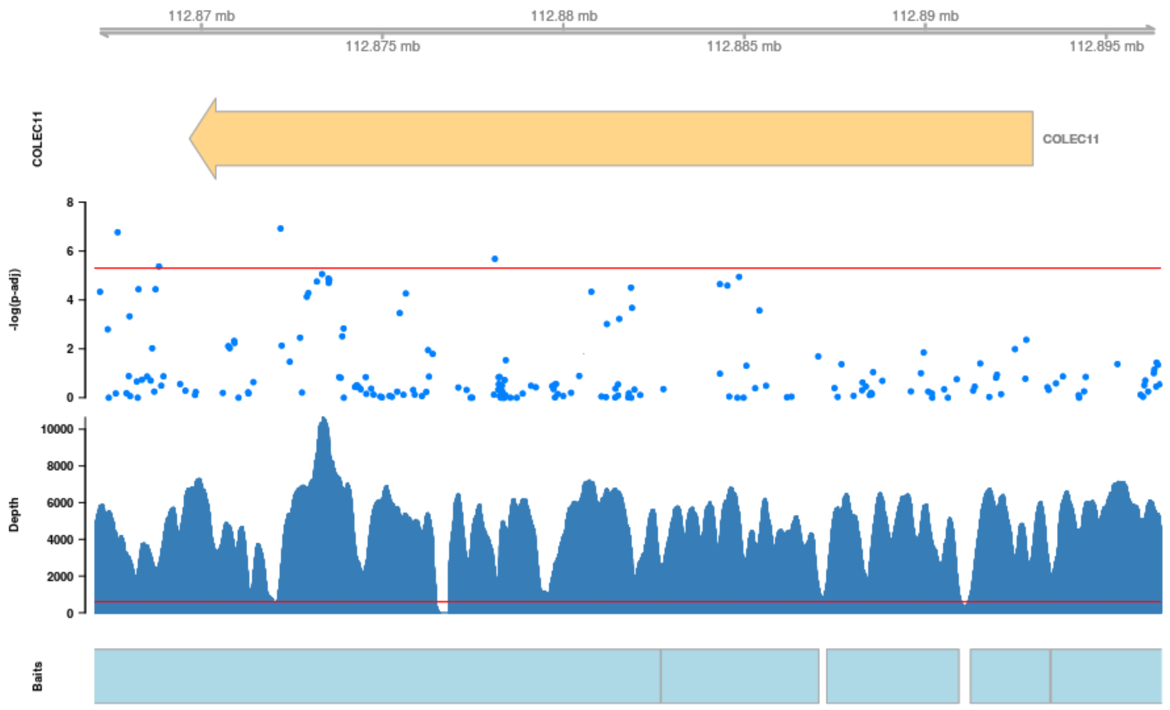
112.87 mb
112.88 mb
112.89 mb
112.875 mb
112.885 mb
112.895 mb
COLEC11
COLEC11
-log(p-adj)
Depth
Baits

c)

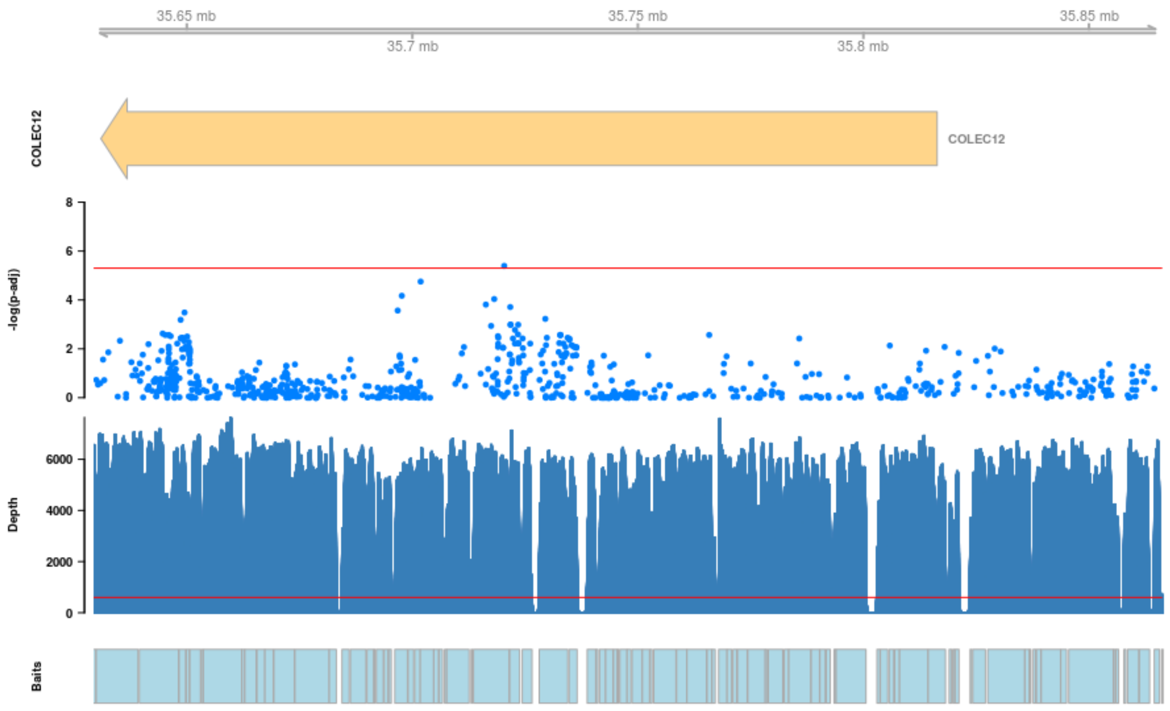

Online Resource 2

Distribution of significant associations are shown for **a** *COLEC11*, **b** *COLEC12*, and **c** *FCN1*. The red line indicates the cutoff for BADGE class II significance ($p < 5 \times 10^{-6}$). The depth of sequencing (total from all pools) and the probes used for target capture are shown to illustrate gaps in sequencing and variant discovery.
