# Supplementary material for: Identification of polymorphisms in the bovine collagenous lectins and their association with infectious diseases in cattle
Source: Immunogenetics. 2018 May 10;70(8):533–46. doi: 10.1007/s00251-018-1061-7 (PMC6061482; doi:10.1007/s00251-018-1061-7)
Supplement: Supplementary file 4 — (PDF 91 kb) [file 251_2018_1061_MOESM4_ESM.pdf]

Electronic Supplementary Data

Identification of polymorphisms in the bovine collagenous lectins and their association with infectious diseases in cattle

Immunogenetics

Russell S. Fraser[1], John S. Lumsden[1,2], Brandon N. Lillie[1]*

[1]Department of Pathobiology, Ontario Veterinary College, University of Guelph

[2]Adjunct Professor, St. George's University, True Blue, Grenada

*Corresponding author: blillie@uoguelph.ca

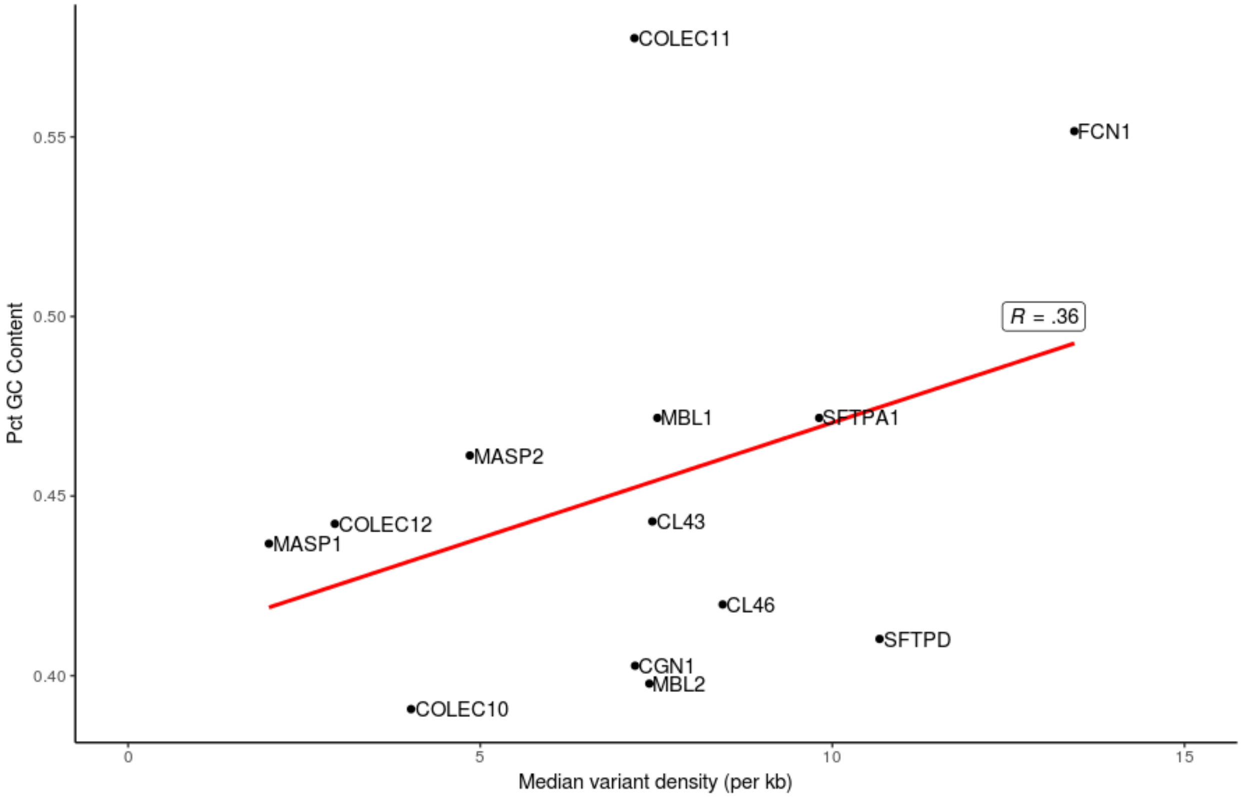

Online Resource 4

The correlation between the percent GC content and the variant density in the targeted genes. Correlation was not significant ($p = 0.23$, Pearson's correlation).
